# Immune checkpoint inhibitors combined with or without radio(chemo)therapy for locally advanced or recurrent/metastatic esophageal squamous cell carcinoma

**DOI:** 10.1007/s12672-023-00783-3

**Published:** 2023-09-04

**Authors:** Xiao-Han Zhao, Hong-Mei Gao, Jing-Yuan Wen, He-Song Wang, Luan-Ying Wu, Chun-Yang Song, Wen-Zhao Deng, Shu-Chai Zhu, Wen-Bin Shen

**Affiliations:** 1 Department of Radiation Oncology, The Forth Hospital of Hebei Medical University, No. 12 Jiankan Road, Chang’an District, Shijiazhuang, 050011 China; 2 Department of Radiation, Shijiazhuang People’s Hospital, Shijiazhuang, China

**Keywords:** Immunotherapy, Radiotherapy, Chemotherapy, Esophageal squamous cell carcinoma, Prognosis, Treatment-related adverse events

## Abstract

**Objective:**

This study was designed to investigate the efficacy and prognostic factors for immune checkpoint inhibitors (ICIs) combined with or without radio(chemo)therapy and to evaluate their toxicity in patients with locally advanced or recurrent/metastatic esophageal squamous cell carcinoma (LA/RM ESCC).

**Methods:**

In this study, 198 patients with locally advanced or recurrent/metastatic (LA/RM) ESCC who received ICIs combined with or without radiotherapy/chemotherapy in the Department of Radiotherapy of the Fourth Hospital of Hebei Medical University were retrospectively analyzed. Univariate and multivariate analyses were performed to determine the prognostic factors for overall survival (OS) and progression free survival (PFS). The factors affecting treatment response and the occurrences of treatment-related adverse events (trAEs) were analyzed.

**Results:**

The median OS and PFS were 30.4 months (95% confidence interval [CI] 15.1–45.7 months) and 15.3 months (95% CI 12.8–17.8 months), respectively. Univariate and multivariate analysis showed that the number of ICI cycles, the intervention of radiotherapy and dysphagia were independent factors affecting OS (Hazard ratio [HR] = 0.39, 2.043 and 0.365, respectively; P = 0.018, 0.001 and 0.032, respectively). The intervention of radiotherapy was an independent factor for PFS (hazard ratio [HR] = 18.149, P = 0.013). The median OS and PFS for patients who had complete response and partial response (Objective response, ORR) were 50.8 months (95% CI 25.8–75.7 months) and 20.5 months (95% CI 14.1–27.0), respectively, which were significantly higher than those in the non-ORR group (OS_non-ORR_:17.5 months, 95% CI 14.0–21.0; χ^2^ = 13.881, *P* < 0.001; PFS_non-ORR_: 12.1 months, 95% CI 10.1–14.1, χ^2^ = 10.676, *P* = 0.001). The intervention of radiotherapy could improve treatment response (χ^2^ = 47.725, *P* = 0.000). In entire study population, 83 patients (41.9%) had ≥ grade 2 trAEs.

**Conclusions:**

ICIs combined with radiotherapy/chemotherapy are safe and effective in LA/RM ESCC patients. Intervention of radiotherapy, the number of immunotherapy cycles and occurrence of dysphagia affecting the overall survival of LR/RM ESCC patients. Intervention of radiotherapy was an independent prognosis factor for OS and PFS and associated with better treatment response.

Esophageal cancer is one of the most common neoplasms in China, whose cases and death toll accounted for > 50% of the total number worldwide [1]. The invasion of esophageal cancer is relatively strong; most patients have lost the opportunity for surgical treatment at the time of clinical diagnosis. While even for patients who received comprehensive therapy, such as radical esophagectomy with or without neoadjuvant therapy, locoregional recurrence or distant metastasis remains the main failure mode after treatment [2, 3]. For patients with a locally advanced stage who have lost the opportunity for surgical resection or refuse to receive surgical treatment and those who experienced treatment failure after surgical resection, radiotherapy with or without chemotherapy remains one of the main treatment modalities; however, its efficacy is far from satisfactory [4, 5].

Recently, the application of immune checkpoint inhibitors (ICIs) has greatly improved the outcomes of patients with esophageal cancer. The ATTRCTION-3, ESCORT, and KEYNOTE-181 studies have confirmed the superiority of ICIs as a second-line treatment for patients with esophageal cancer [6–8]. The results of the KEYNOTE-590 and ESCORT studies have shown that the usage of ICIs as first-line treatment can significantly improve the overall survival (OS) and progression-free survival (PFS) for patients with esophageal cancer [9, 10]. Furthermore, the application of ICIs as neoadjuvant modalities for patients with locally advanced resectable esophageal cancer has improved the pathological complete response rate and R0 resection rate [11]. In summary, increasing pieces of evidence have shown that ICI monotherapy and ICI-based combination therapy can significantly improve the prognosis of patients with esophageal cancer.

At present, many studies have focused on the advantages of ICIs combined with radiotherapy [12, 13]; however, reports on the clinical practice of ICIs combined with radiotherapy with or without chemotherapy in treating patients with advanced or recurrent/metastatic (LA/RM) ESCC are few. To further observe and verify the efficacy and safety of ICIs combined with radiotherapy/chemotherapy, we conducted this real-world retrospective analysis study and chose 3-year OS as the primary outcome.

## Materials and methods

Our study included patients who met the following inclusion criteria:

1. age ≥ 18 years; (2) histologically confirmed diagnosis of ESCC; (3) the presence of at least one measurable lesion based on the RECIST 1.1 criteria before treatment initiation; (4) the absence of serious infections or other severe systemic diseases before treatment; (5) receipt of at least three cycles of ICI therapy; and (6) receipt of ICIs as either first- or second-line therapy, without previous exposure to immunotherapy for patients receiving ICIs as a second-line treatment. Patients were excluded from the study if they met any of the following criteria: (1) coexistence of other malignancies aside from ESCC; (2) abnormal bone marrow hyperplasia or other hematopoietic system diseases before treatment; (3) active infections requiring intervention, such as human immunodeficiency virus or viral hepatitis infection, before treatment; and (4) need for drug intervention due to autoimmune diseases or other severe systemic diseases.
2. Data collection and outcome assessment: Clinical data were collected mainly through the patients’ clinical medical records and telephone follow-up, including baseline clinical characteristics, demographic background, peripheral blood biochemical data, treatment modalities, treatment response, and safety. Treatment-related adverse events (trAEs) were applied to evaluate the associated adverse effects. The Immune-Related Response Evaluation Criteria In Solid Tumors (irRECIST) was used to evaluate the efficacy of immunotherapy. To avoid pseudoprogression from immunotherapy, treatment response was evaluated 3 months after treatment initiation, including complete response (CR), partial response (PR), stable disease (SD), and progressive disease (PD). The overall response rate (ORR) was calculated based on the percentage of patients who achieved CR and PR. The disease control rate (DCR) was calculated based on the percentage of patients who achieved CR, PR, and SD. The severity of trAEs was assessed according to the National Cancer Institute Common Terminology Criteria for Adverse Events, version 5.0 (only toxicities ≥ grade 2 were analyzed in this study). Treatment failure included local regional recurrence (LRR) and distant metastasis (DM); LRR was defined as failure in the local regional area, including primary tumor and adjacent lymph node recurrence; DM was defined as nonregional lymph node metastasis or metastasis to any distant organ. The TNM stage was classified according to the 8th edition of the International Union Against Cancer classification. Oligometastasis was defined as ≤ 3 metastases within in organ or 1 distant lymph node metastasis. All patients were informed of the treatment protocol before the treatment began. This study was approved by the Ethics Committee of the Fourth Hospital of Hebei Medical University.
3. Radiotherapy: Overall, 136 patients received radiotherapy. All patients received involved-field irradiation. According to the range of radiotherapy target, the patients were divided into the “radiotherapy for all lesions” (RAL) group and the “radiotherapy for partial lesions” (RPL) group. The RAL means all visible tumors were included in the radiotherapy target, while the RPL means only part of visible tumors were included in radiotherapy target. The RAL group consisted of 83 (61.0%) patients, whereas the RPL group comprised 53 (39.0%) patients. Furthermore, 101 patients received conventional fractions with radical intent (50–60 Gy/25–30 fractions) and 35 patients received palliative radiotherapy (30–48 Gy/10–24 fractions), including 41 lesions. The median biological equivalent dose (α/β = 10 Gy) was 56 Gy.
4. Follow-up: All patients were regularly examined and followed up. The routine examinations included blood tests, chest and abdominal computed tomography scan, esophageal barium examination, and superficial lymph node ultrasonography. The patients were followed up every 2–3 cycles during immunotherapy and every 1–3 months after the end of immunotherapy. The last follow-up date was December 31, 2022.
5. Statistical analysis: Statistical Package for the Social Sciences (version 25.0; IBM Corp, Armonk, NY, USA) was used for all statistical analyses. The chi-square test or Fisher’s exact test was applied to analyze the difference in categorical variables. Meanwhile, the Mann–Whitney U test was used to compare continuous variables. The Kaplan–Meier method was used to estimate the OS and PFS. The log-rank test was used for univariate stratified comparison, variables with *P* < 0.10 in the univariate analysis were included in the multivariate Cox proportional hazards regression model. The primary endpoints were OS and PFS. OS was defined as the time from the start of treatment to the date of death or the last follow-up. PFS was defined as the time from the start of treatment to the first documented disease progression, death, or last contact, whichever occurred first.

## Results

### Clinical characteristics

This study retrospectively analyzed consecutive eligible patients with ESCC who received ICI treatment in the Department of Radiotherapy of the Fourth Hospital of Hebei Medical University from January 2018 to January 2021. The last follow-up was on December 31, 2022, a total of 198 patients were recruited, with a median follow-up duration of 30.5 months (95% CI 28.3–32.7 months). Of the 198 patients, 33 has dysphagia, 77 patients were treated with ICI therapy as the first-line treatment, and 121 patients were treated with ICI therapy as the second-line treatment. A total of 133 patients received ICI and radiotherapy in the same line, of whom 47 received concurrent treatment of ICIs and radiotherapy, which means the 21-day cycle of ICIs continued during the time of radiotherapy. 86 patients received sequential ICIs and radiotherapy, which means ICIs was applied every 21 days and the cycles of ICIs would stop during the time of radiotherapy. Those treated with ICI therapy as the first-line treatment were staged according to the eighth edition of the American Joint Committee on Cancer esophageal cancer staging criteria, of whom 64.9% were stage IV, and lymph node metastasis was most common. Most patients (87.6%) receiving ICI therapy as the second-line treatment experienced LRR after the first-line treatment, among these patients, the fist-line treatment modalities were radiotherapy and chemotherapy (43.8%) and neoadjuvant chemotherapy followed by surgery (24.8%). Overall, 136 patients received radiotherapy, including 83 (61.0%) in the RAL group and 53 (39.0%) in the RPL group. Most patients (81.8%) received chemotherapy. The ICIs + CRT group accounted for 59.1% of the entire cohort. See Tables 1 and 2 for details.

**Table 1:** Univariate and multivariate analysis of all esophageal squamous cell carcinoma patients receiving immune checkpoint inhibitors therapy

Index (The number of patients)	N (%)
Gender: male/female (n = 198)	143(72.2)/55(27.8)
Age (years): median value/range (n = 198)	64/39 ~ 85
ECOG score: 0/1/2 (n = 198)	22(11.1)/90(45.5)/86(43.4)
Primary lesion location: cervical/upper thoracic/middle thoracic/lower thoracic (n = 198)	13(6.6)/41(20.7)/88(44.4)/56(28.3)
Degree of differentiation: undifferentiated or poorly differentiated/moderately differentiated/well differentiated (n = 198)	53(26.8)/94(47.5)/51(25.8)
Initial clinical TNM staging: II/III/IV (n = 77)	10(13.0)/17(22.1)/50(64.9)
Type of second-line failure: LRR/DM/ both (n = 121)	57(47.1)/15(12.4)/49(40.5)
Initial treatment modality: RT/CRT/S/nCRT + S/nCT + S (n = 121)	15(12.4)/53(43.8)/10(8.3)/13(10.7)/30(24.8)
Dysphagia: Yes/no (n = 198)	33(16.7)/165(83.3)
Utility of Immunotherapy: first-line/second-line (n = 198)	77(38.9)/121(61.1)
Immune drugs: Camrelizumab/Sintilimab/Pembrolizumab/Toripalimab/Tislelizumab (n = 198)	109(55.1)/31(15.7)/30(15.2)/18(9.1)/10(5.1)
Number of immunotherapy cycles: range/median/ < 5 cycles / ≥ 5 cycles (n = 198)	3 ~ 43/4/101(51.0)/97(49.0)
Utility of Chemotherapy: Yes/No (n = 198)	162(81.8)/36(18.2)
Number of chemotherapy cycles: range/median/platinum plus paclitaxel/platinum plus other/single agent (n = 162)	2 ~ 12/4/98(60.5)/37(22.8)/27(16.7)
Utility of radiotherapy: Yes/No (n = 198)	136(68.7)/62(31.3)
Coverage of radiotherapy: RPL/RAL (n = 136)	53(39.0)/83(61.0)
modality of ICIs-combination therapy: ICIs + CRT/ICIs + CT/ICIs + RT/ ICIs alone (n = 198)	117(59.1)/45(22.7)/19(9.6)/17(8.6)

ECOG: eastern cooperative oncology group; ICIs: immune checkpoint inhibitors; LRR: Locoregional recurrence; DM: distant metastasis; S: surgery; CRT: chemoradiotherapy; CT: chemotherapy; RT: radiotherapy; nCRT + S: neoadjuvant chemoradiotherapy plus surgery; nCT + S: neoadjuvant chemotherapy plus surgery; RPL: Radiotherapy For Partial Lesions; RAL: Radiotherapy for All Lesions

**Table 2:** Analysis of recurrent/ metastasis sites for esophageal squamous cell carcinoma patients receiving first or second line immune checkpoint inhibitors therapy

Failure sites for patients receiving first-line therapy (77 cases)	N (%)	Failure sites for patients receiving second-line therapy (121 cases)	N (%)
Regional lymph nodes metastasis	18 (23.4)	Primary esophageal lesion/postoperative tumor bed area	26 (21.5)
Distant lymph node metastasis	7 (9.1)	Regional lymph nodes metastasis	19 (15.7)
Regional and distant lymph node metastasis	37 (48.1)	Primary esophageal lesion/postoperative tumor bed + locoregional lymph nodes	12 (9.9)
Organ metastasis	2 (2.6)	Distant lymph node metastasis	6 (5.0)
Organs and regional lymph nodes metastasis	3 (3.9)	organs metastasis	9 (7.4)
Organ and distant lymph node metastasis	1 (1.3)	Regional and distant lymph node metastasis	20 (16.5)
None	9 (11.7)	Organs and lymph node metastasis	29 (24.0)

### Prognosis analysis

**Fig. 1:**
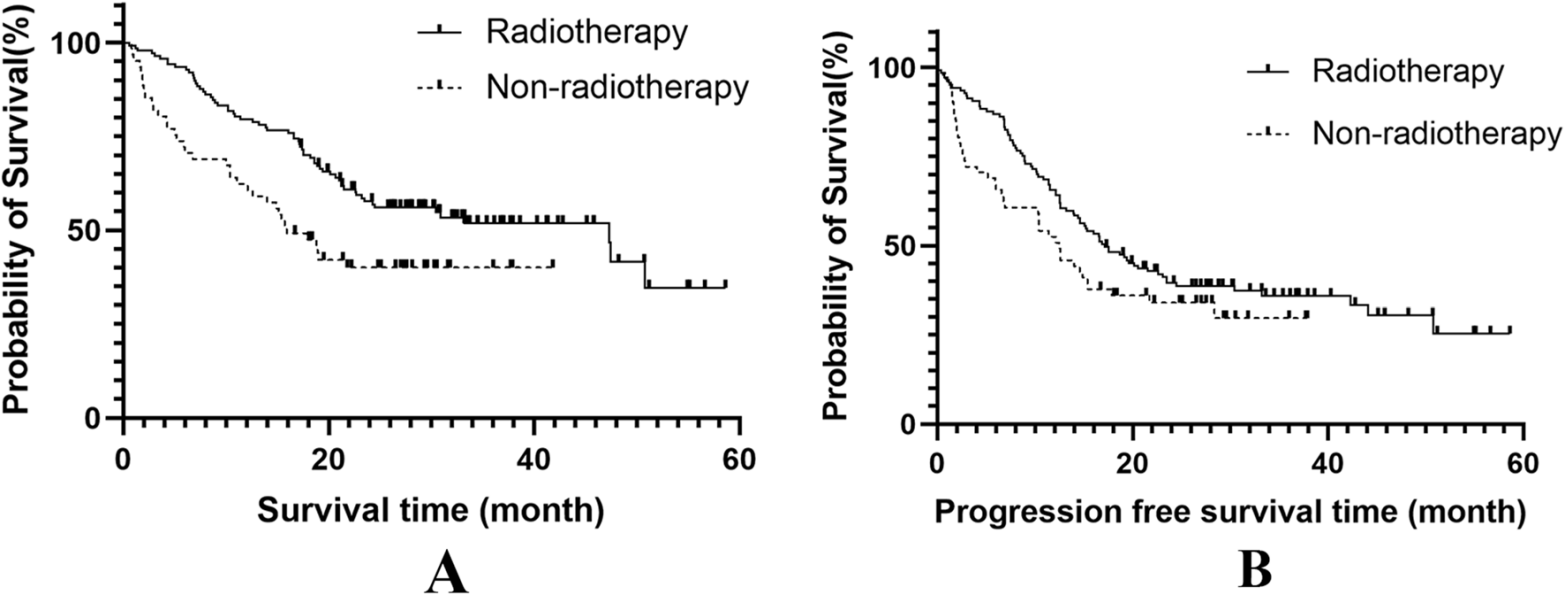
The survival curve of receiving radiotherapy group and receiving non-radiotherapy group (**A** Overall survival, *P* = 0.016; **B** Progression free survival, *P* = 0.023)

The 1-, 2-, and 3-year OS and PFS rates were 74.2%, 52.2%, and 47.6% and 61.6%, 37.6%, and 34.0%, respectively. The median OS and PFS were 30.4 months (95% CI 15.1–45.7 months) and 15.3 months (95% CI: 12.8–17.8 months), respectively. The results of the univariate analysis showed that lower Eastern Cooperative Oncology Group (ECOG) scores, receiving ICI therapy as the first-line treatment modality, metastasis type, receiving radiotherapy, coverage of radiotherapy, receiving ICIs for ≥ 5 cycles, ICIs-related adverse events, dysphagia, receiving chemotherapy, were significant impact factors for OS (P = 0.006, 0.003, 0.024, 0.016, 0.000, 0.009, 0.018, 0.017, 0.059 respectively). Additionally, ECOG socres, dysphagia, receiving radiotherapy, ICIs-related adverse events, coverage of radiotherapy were significant impact factor for PFS (P = 0.023, 0.034, 0.045, 0.064 and 0.009, respectively). See Table 3 for further details. The Survival curve for radiotherapy group and non-radiotherapy group were showed in (Fig. 1A, B).

**Table 3:** Univariate analysis of OS and PFS for the whole cohort

Index	N	OS rate (%)			Median (month)	*P*	PFS rate (%)			Median (month)	*P*
		1-year	2-year	3-year			1-year	2-year	3-year		
Gender						0.215					0.153
Male	143	71.3	48.6	43.8	23.3		58.7	34.2	30.6	14.6	
Female	55	81.8	61.6	58.0	47.4		69.1	46.6	42.3	22.6	
Age (years)						0.816					0.761
≤ 64	104	72.1	54.3	49.5	30.9		61.5	39.2	35.1	14.9	
> 64	94	76.6	49.7	48.4	23.5		61.7	35.5	32.3	15.4	
ECOG score						0.006*					0.023*
0–1	112	79.5	60.2	58.3	47.3		67.9	44.0	40.8	17.9	
2	86	67.4	41.6	33.8	21.0		53.5	29.3	25.4	12.5	
Primary lesion location						0.513					0.700
Cervical/upper	54	70.4	46.7	46.7	23.2	0.771	61.6	31.9	28.3	14.4	0.627
Thoracic/middle	88	80.7	58.5	52.2	47.3	0.270	65.9	40.4	36.8	16.5	0.767
Thoracic/lower	56	67.9	47.4	41.9	21.2	Reference	55.4	38.6	34.3	12.6	Reference
Degree of differentiation						0.264					0.749
Undifferentiated/poorly	53	79.2	59.6	59.6	47.3	0.110	60.4	39.3	39.3	16.0	0.586
Moderately differentiated	94	74.5	52.3	45.4	24.5	0.266	63.8	37.4	33.7	16.6	0.457
Well differentiated	51	68.6	44.1	40.4	21.5	Reference	58.8	36.4	28.7	14.0	Reference
Clinical TNM stage						0.117					0.256
II + III	79	79.7	60	52.1	47.3		67.1	43.4	36.0	19.1	
IV	119	70.6	47	44.3	21.7		58.0	33.8	32.3	14.6	
Treatment lines						0.003*					0.162
First line	77	85.7	63.3	57.7	–		68.8	39.8	38.2	16.6	
Second line	121	65.3	45.2	41.5	20.1		57.0	36.3	31.1	14.0	
Metastasis type						0.024*					0.108
Lymph node metastasis	44	81.8	53.5	–	–	0.689	65.9	40.6	40.6	16.6	0.826
Organic metastasis	59	61	36.3	–	15.9	0.094	50.8	24.2	24.2	12.6	0.196
Lymph node and organic metastasis	11	63.6	–	–	–	Reference	54.5	36.4	36.4	28	Reference
Number of metastases						0.562					0.646
Oligometastasis	69	72.5	47.2	47.2	22.7		62.3	34.1	31.4	15.4	
Multiple metastasis	45	66.7	40.1	40.1	21.3		53.3	32.8	32.8	12.5	
Baseline lymphocyte count						0.679					0.949
< 1.0 × 10^9^	88	77.3	49.1	45.0	23.5		58.2	37.5	36.4	14.9	
≥ 1.0 × 10^9^	110	71.8	54.7	50.0	47.3		65.9	37.8	31.8	16.9	
Dysphagia						0.017*					0.034*
Yes	33	66.7	30.3	26.5	18.2		51.5	21.2	18.2	14.0	
No	165	75.8	56.8	52.6	47.4		63.6	41.0	37.8	16.5	
Chemotherapy						0.059*					0.167
Yes	162	75.9	55.4	49.8	33.2		63.0	39.3	35.8	16.6	
No	36	66.7	37.5	37.5	15.9		55.6	25.3	25.3	12.6	
Chemotherapy modality						0.590					0.596
Single agent	36	83.3	51.4	39.6	30.4		66.7	49.8	34.6	20.1	
Doublet agent	126	73.8	55.6	54.7	47.3		61.9	36.2	36.2	15.4	
Number of chemotherapy cycles						0.178					0.671
< 4	74	68.9	48.2	44.1	22.6		60.8	37.2	33.0	16.9	
≥ 4	88	81.8	61.7	54.7	47.3		59.5	41.4	37.8	15.5	
Radiotherapy						0.016*					0.023
Yes	136	78.7	56.6	50.6	47.4		64.7	38.2	34.5	16.6	
No	62	64.5	42.7	42.7	17.9		54.8	36.7	33.0	12.6	
Coverage of radiotherapy						0.000*					0.009*
RAL	83	81.5	61.9	60.1	–		66.9	42.5	41.1	17.5	
RPL	53	62.2	36.0	28.4	17.4		52.7	29.5	23.8	12.5	
Number of immunotherapy cycles						0.009*					0.139
< 5 cycles	101	62.4	43.0	43.0	19.1		55.4	34.2	34.2	12.6	
≥ 5cycles	97	86.6	61.7	52.1	47.3		68.0	41.3	33.9	17.5	
ICIs-related adverse events											
Grade 1–2	74	82.4	58,0	47.3	30.9	0.018*	70.3	39.0	29.5	17.8	0.064*
Grade 3–4	27	51.9	35.7	31.2	17.4		37.0	25.4	–	8.5	

ECOG: eastern cooperative oncology group; ICIs: immune checkpoint inhibitors; CRT: chemoradiotherapy; CT: chemotherapy; RT: radiotherapy; *: *P* < 0.05

### Treatment response

**Fig. 2:**
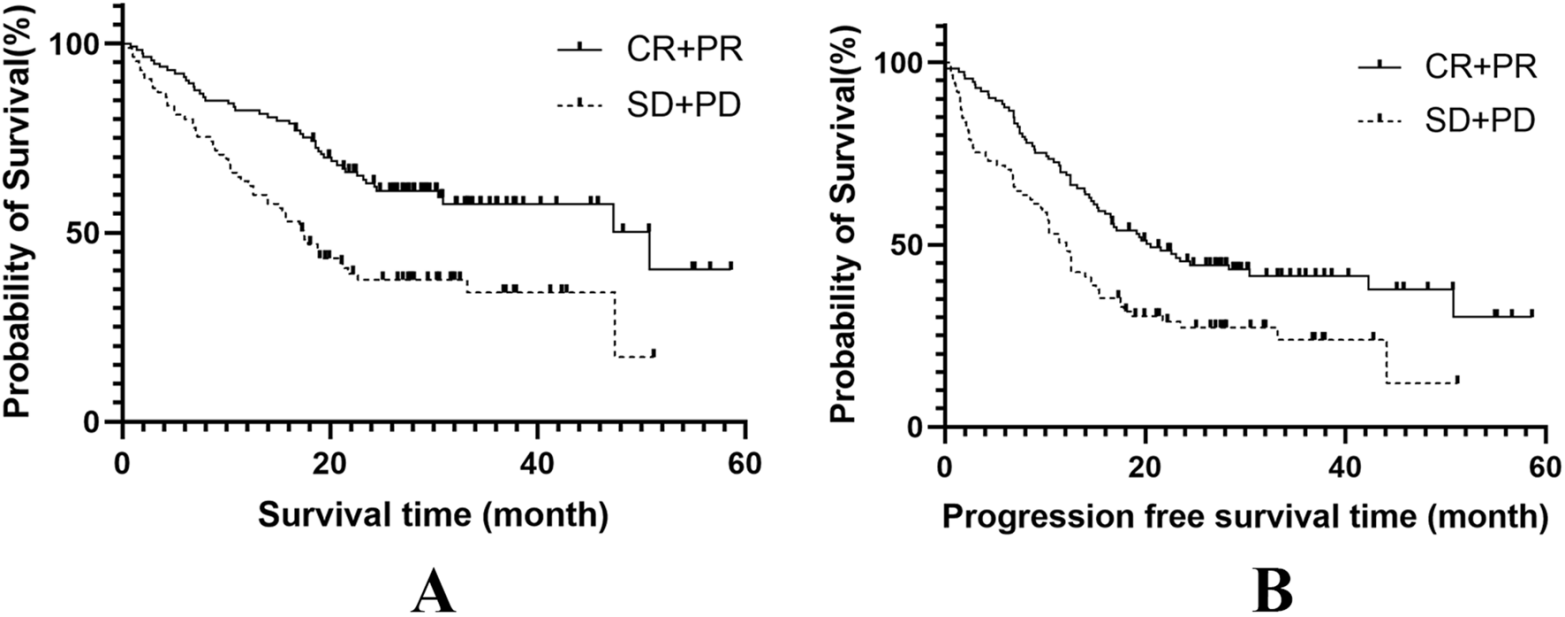
The survival curve of complete response/partial response group (CR + PR) group and stable disease/progression disease (SD + PD) group (**A** Overall survival, P < 0.001; **B** Progression free survival,* P* = 0.001)

Of the entire cohort, seven (3.5%), 106 (53.5%), 75 (37.9%), and 10 (5.1%) patients achieved CR, PR, SD, and PD, respectively. The ORR was 57.1% (113/198), and the DCR was 94.9% (188/198). The 1-, 2-, and 3-year OS and PFS rates for the 113 patients in the ORR group were 82.3%, 63.1%, and 57.5%, respectively, and 69.9%, 45.4%, and 41.4%, respectively. The median OS and PFS rates were 50.8 months (95% CI 25.8–75.7 months) and 20.5 months (95% CI 14.1–27.0 months), respectively. The 1-, 2-, and 3-year OS and PFS rates for the 85 patients in the non-ORR group were 63.5%, 37.6%, and 34.2%, respectively, and 50.6%, 27.3%, and 23.8%, respectively. The median OS and PFS rates were 17.5 months (95% CI 14.0–21.0 months) and 12.1 months (95% CI 10.1–14.1 months), respectively. Significant differences in OS (χ^2^ = 13.881; *P* < 0.001) and PFS (χ^2^ = 10.676; *P* = 0.001) rates were observed between the two groups. See (Fig. 2A, B).

Then, we analyzed the efficacy of different treatment modalities. The results showed a significant difference in the efficacy among the four treatment modalities (i.e., ICIs + CRT, ICIs + RT, ICIs + CT, and ICIs alone) (χ^2^ = 47.725; *P* = 0.000). From Table 4, it can be found that the addition of radiotherapy can improve the treatment response.

**Table 4:** Analysis of treatment efficacy according to the modality of ICIs-combination therapy

ICIs-combination modality	N	Treatment response (%)				χ^2^	*P*
		CR	PR	SD	PD		
ICIs + CRT	117	5 (4.3)	78 (66.7)	28 (23.9)	6 (5.1)	47.725	0.000
ICIs + RT	19	2 (10.5)	11 (57.9)	4 (21.1)	2 (10.5)		
ICIs + CT	45	0 (0)	14 (31.1)	31 (68.9)	0 (0)		
ICIs	17	0 (0)	3 (17.6)	12 (70.6)	2 (11.8)		

ICIs: Immune checkpoint inhibitors; CRT: Chemoradiotherapy; CT: Chemotherapy; RT: Radiotherapy; CR: Complete Response; PR: Partial Response; SD: Stable disease; PD: Progression disease

### Multivariate analysis of prognosis factors

The factors with P < 0.1 in the univariate analysis were included in the COX multivariate analysis model. The results showed that the number of ICI cycles, the application of radiotherapy and dysphagia were independent factors affecting OS rate (hazard ratio [HR] = 0.39, 2.043 and 0.365, respectively; P = 0.018, 0.001 and 0.032, respectively). The application of radiotherapy was an independent factor for PFS (hazard ratio [HR] = 18.149, P = 0.013) See Table 5 for details.

**Table 5:** Multivariate analysis affecting overall survival and progression free survival

Factor	Subgroup	Regression coefficient	Standard error	*P value*	HB	95% CI	
						Inferior limit	Upper limit
OS							
Number of ICIs cycles	< 5 cycles/ ≥ 5 cycles	− 0.941	0.398	0.018*	0.390	0.179	0.852
ECOG socres	0–1/2	− 0.080	0.527	0.880	0.924	0.329	2.592
Radiotherapy	Yes/No	3.233	1.490	0.030*	25.365	1.350	3.092
Dysphagia	Yes /No	− 1.007	0.471	0.032*	0.365	0.145	0.918
Metastasis type				0.417			
	Lymph node metastasis/ Lymph node and organic metastasis	8.236	90.545	0.928	3773.583	0.000	4.455E + 80
	Organic metastasis/ Lymph node and organic metastasis	8.922	90.545	0.922	7494.204	0.000	8.845E + 80
Chemotherapy	Yes/No	0.056	0.616	0.927	1.058	0.316	3.539
ICIs-related adverse events	Grade 1–2/Grade 3–4	0.261	0.469	0.577	1.299	9.518	3.255
Coverage of radiotherapy	RAL/RPL	0.061	0.486	0.901	1.063	0.410	2.755
Treatment lines	First line/Second line	0.521	0.482	0.281	1.683	0.654	4.333
PFS							
ECOG socres	0–1/2	0.472	0.313	0.132	1.604	0.868	2.964
Dysphagia	Yes /No	− 0.661	0.372	0.076	516	0.249	1.071
Radiotherapy	Yes/No	2.899	1.172	0.013*	18.149	1.825	180.499
ICIs-related adverse events	Grade 1–2/Grade 3–4	− 0.012	0.328	0.970	0.988	0.519	1.880
Coverage of radiotherapy	RAL/RPL	0.055	0.319	0.863	1.056	1.056	1.056

ICIs: Immune checkpoint inhibitors; CRT: Chemoradiotherapy; CT: Chemotherapy; RT: Radiotherapy; CR: Complete Response; PR: Partial Response; SD: Stable disease; PD: Progression disease; RPL: Radiotherapy For Partial Lesions; RAL: Radiotherapy for All Lesions; *: *P* < 0.05

### Analysis of trAEs

Eighty-three patients (41.9%) experienced ≥ grade 2 trAEs in the entire cohort; the incidence of ≥ grade 2 trAEs rate in the ICIs+CRT, ICIs+CT, ICIs+RT, and ICIs group was 45.3% (53–117), respectively. By the chi-square test, no significant difference in the incidence of trAEs was observed among the different treatment modalities (χ^2^ = 1.758; *P* = 0.624). The incidence of ≥ grade 2 trAEs in patients who received ICIs as the first-line treatment and those who received ICIs as the second-line treatment was 40.3% (31/77) and 43.0% (52/121), respectively, whose difference was not statistically significant (χ^2^ = 0.143; *P* = 0.706).

Among the 83 patients, 70 experienced grade 2 trAEs. The most common trAE was myelosuppression (28 cases, 14.1%), followed by gastrointestinal reactions (21 cases, 10.6%); pneumonia (15 cases, 7.6%); esophagitis (13 cases, 6.6%); abnormal thyroid function (9 cases, 4.5%); and liver function damage (4 cases, 2.0%). Thirteen patients had grade 3/4 trAEs (6.6%): esophageal fistula in 6 cases, myelosuppression in 4 cases, pneumonitis in 2 cases, and liver dysfunction in 1 case.

## Discussion

Based on the results of currently available phase III randomized clinical trials, ICIs combined with chemotherapy rather than chemotherapy alone have been recommended as the first-line treatment for advanced esophageal cancer. In patients with locally advanced or relapsing/metastatic (LA/RM) ESCC, the addition of radiotherapy to ICIs and chemotherapy has become a new strategy, which may have synergistic effects and better efficacy; however, studies that focused on these triple modalities for LA/RM ESCC are few [14–16]. To explore the efficacy of ICI combination modalities in the real world, a retrospective analysis of 198 LA/RM ESCC cases was performed in this study.

According to the literature, this should be the study with the largest number of LA/RM ESCC cases treated with ICIs combined with radiotherapy or chemotherapy and with a follow-up duration of 3 years. The OS and PFS rates of these patients were 30.4 months (95% CI 15.1–45.7 months) and 15.3 months (95% CI 12.8–17.8 months), respectively. Zhang et al. [17] conducted a prospective study involving 20 patients with esophageal cancer who received chemoradiotherapy combined with camrelizumab, and the 1- and 2-year OS and PFS rates were 85.0% and 69.6%, respectively, and 80.0% and 65.0%, respectively. The median OS and PFS rates were 16.7 months (95% CI 5.9–27.9 months) and 11.7 months (95% CI 0–30.3 months), respectively. Park et al. [18] examined a group of patients with esophageal cancer who received chemoradiotherapy combined with dual immunotherapy (durvalumab and tremelimumab), and the 2-year OS and PFS rates of the patients were 75% and 57.5%, respectively. Other relevant meta-analysis studies have also confirmed the effectiveness of radiotherapy/chemoradiotherapy combined with ICIs in treating patients with esophageal cancer. For example, Wu et al. [19] conducted a meta-analysis involving 668 patients with esophageal cancer who received radiotherapy or chemoradiotherapy combined with ICIs, and the 1- and 2-year OS rates were 84.5% and 68.3%, respectively. Compared with those studies, the 1- and 2-year OS or PFS rates in this study were slightly lower, which was considered to be related to the inclusion of many patients with stage IV disease and those treated after the failure of the first-line treatment. Wu et al. [14] retrospectively analyzed the treatment of 127 patients with metastatic or recurrent ESCC, of whom 40 received radiotherapy combined with ICIs, and the median OS and PFS rates of these patients were 11.9 months and 5.45 months, respectively. Radiotherapy–ICI combination therapy could increase the OS rates of patients with local recurrence (*P* = 0.026). Wang et al. [20] evaluated 50 patients with advanced esophageal cancer treated with sintilimab, of whom 38 had esophageal or regional lymph node metastasis and 24 received radiotherapy. The results showed that the 1-year OS and PFS rates of the entire group were 67.1% and 49.2%, respectively. The median PFS was 11.3 months (95% CI 5.0–17.6 months). The ORR and DCR were 60% (30/50) and 92% (46/50), respectively. The 1-year OS rate of the patients receiving ICIs combined with radiotherapy was higher than that of patients receiving ICIs without radiotherapy (85.9% *vs.* 53.2%; *P* = 0.020). The results of the aforementioned studies suggest that the combination of ICIs and chemoradiotherapy can improve survival compared with conventional chemoradiotherapy alone in ESCC.

Treatment response is the main prognostic factor for patients with ESCA, which has been confirmed by previous clinical studies [22, 23]. For patients receiving neoadjuvant therapy, the main indicators to judge the treatment response were the pathological CR rate and major pathological response rate, whereas, for patients receiving immunotherapy, treatment response evaluation was mainly based on the irRECIST. This study illustrated that the treatment response of immunotherapy combined with radiotherapy was an independent factor for predicting the OS and PFS rates. The intervention of radiotherapy significantly improved prognosis. At present, no direct evidence exists to show the superiority of ICIs + CRT; however, some small-sample prospective studies on preoperative ICIs + CRT as a neoadjuvant therapy for patients with esophageal cancer have been conducted. The results of real-world and retrospective studies have also proved the superiority of immunotherapy. This point was also confirmed by the study by Wei et al. [21]. After a retrospective analysis of 48 patients with recurrent or metastatic ESCC, they found that the median PFS of 29 patients receiving ICIs + CRT and 19 patients receiving ICIs + CT were 6.0 months and 5.0 months, respectively (*P* = 0.025). The 6-month OS rates were 70.8% and 47.9%, respectively (*P* < 0.001). Yang et al. [22] performed a retrospective analysis of 196 patients with esophageal cancer, and the results showed that the median PFS (8.5 *vs.* 3.2 months, *P* < 0.001) and OS (18.9 *vs.* 9.8 months, *P* = 0.010) in the immune-combined therapy group were significantly higher than those in the immune-alone treatment group. The relationship between the duration of immunotherapy and prognosis is also one of the hot issues that clinicians pay attention to. In several phase III clinical studies, the duration of ICI treatment is usually no more than 2 years or the disease progresses or the patient cannot tolerate it. The PACIFIC study [23] showed that among patients with non-small-cell lung cancer who received concurrent chemoradiotherapy, those who received maintenance therapy with durvalumab for 1 year had a significant benefit compared with those who did not receive durvalumab. However, the CheckMate-153 trial [24] showed that patients treated with nivolumab for > 1 year had significantly better OS and PFS than those treated with fixed-dose nivolumab alone for 1 year. The generation of memory T lymphocytes in the tumor microenvironment is thought to contribute to sustained responses to ICI treatment. To identify markers that can predict optimal treatment duration with ICIs, further research into the mechanisms behind ICI treatment is necessary. Interestingly, this study found that patients who received at least five cycles of immunotherapy had better OS.

Currently, research on the significance of local radiotherapy for patients receiving systemic therapy and the relationship between the number of irradiation sites and prognosis for LA/RM ESCC is limited. Several studies on lung cancer have shown that simultaneous irradiation of the primary lesion and oligometastatic lesions can improve patient prognosis [25, 26]. The Canadian SABR-COMET study released its 8-year follow-up results [27], which showed that the 8-year OS rates for the stereotactic ablative radiotherapy to all metastases group and the palliative standard-of-care treatment group were 27.2% and 13.6% (*P* = 0.008), respectively, and the 8-year PFS rates were 21.3% and 0.0% (*P* < 0.001), respectively. The addition of local therapy to systemic therapy can effectively improve the prognosis of patients, which may be related to the following mechanisms: first, local treatment can reduce the burden of tumor-resistant cells after systemic treatment, and the early addition of local treatment can reduce the occurrence of distant micrometastatic lesions during treatment [28, 29]; second, local treatment can make irradiated tumor cells more sensitive to systemic medicine therapy; third, by reducing the load of tumor cells, local treatment can alleviate the growth of distant micrometastatic lesions [30].

trAEs often appear as the treatment progresses, and the occurrence and severity of trAEs can be influenced by different treatment regimens, the sequence in which treatments are applied, patient age, and individual differences in physical conditions. Studies have shown that most patients experience at least one trAE of any grade during immunotherapy treatment [31]. Because grade 1 trAEs do not significantly affect patient treatment, this study analyzed grade ≥ 2 trAEs. In our study, we observed that the addition of radiotherapy did not significantly increase the incidence or severity of trAEs in patients, except for some hematological complications. The incidence of grade ≥ 2 trAEs, including pneumonia and esophagitis, was similar to the results of previous studies on the combination therapy of ICIs for advanced ESCC [9, 10, 17]. The most severe trAE observed in our study was esophageal fistula (6 cases, 3.0%), which is similar to the findings of Zhang et al. [17], who reported that two of 19 patients (10.5%) had esophageal fistula. The incidence of esophageal fistula in patients with esophageal cancer receiving curative radiotherapy with or without chemotherapy ranged from 5.6% to 33% [32, 33]. The main risk factors for esophageal fistula occurrence were patient age, ECOG score, body mass index, advanced esophageal cancer stage, poor treatment response, combination of chemotherapy/radiotherapy, ulcerative esophageal cancer, and repeat radiation. Among the six patients with esophageal fistula in our study, four received ICIs combined with chemoradiotherapy as the first-line treatment (3.3%), one received ICIs combined with chemotherapy (2.6%), and one received ICIs alone. Therefore, clinicians should be cautious of the occurrence of esophageal fistula in patients with esophageal cancer who have the aforementioned risk factors and those receiving ICIs combined with chemotherapy/radiotherapy.

The limitations of this study were as follows: first, this study adopted a single-center retrospective study design, which carries the inherent risks of selection bias and misclassification bias. Second, the patients or their family members may have recall bias regarding the treatment modalities and time of death during follow-up; some errors may have occurred. Third, the treatment regimen was mainly determined by the physician; thus, there may be inconsistencies in the drug and radiotherapy doses, and this study did not evaluate whether pretreatment PD-L1 expression levels would affect the patients’ prognosis. Therefore, the results of this study should be confirmed by prospective multicenter studies with larger sample sizes and longer follow-up durations.

In summary, our study demonstrated that ICIs combined with radiotherapy/chemotherapy are safe and effective for LR/RM ESCC. The number of immunotherapy cycles, the occurrence of dysphagia and the intervention of radiotherapy are the main factors affecting overall survival for LR/RM ESCC patients. Intervention of radiotherapy was an independent prognosis factor for OS and PFS and associated with bettter treatment response. While the results of this study should be confirmed by conducting a multicenter, prospective study with a larger sample size and longer follow-up duration.

## Data Availability

The datasets used or analyzed during the current study are available from the corresponding authors on reasonable request.
